# Serum Lipids and Cardiovascular Disease Mortality in Iranian Population: Joint Modeling of Longitudinal and Survival Data in Tehran Lipid and Glucose Study (TLGS) Cohort

**DOI:** 10.31661/gmj.v8i0.1516

**Published:** 2019-09-18

**Authors:** Bagher Pahlavanzade, Farid Zayeri, Taban Baghfalaki, Farzad Hadaeg, Davood Khalili, Mohammad Shoaib Hamrah, Edwin Paul, Fereidoun Azizi, Alireza Abadi

**Affiliations:** ^1^Departments of Biostatistics, School of Allied Medical Sciences, Shahid Beheshti University of Medical Sciences, Tehran, Iran; ^2^Departments of Statistics, Faculty of Mathematical Sciences, Tarbiat Modares University, Tehran, Iran; ^3^Prevention of Metabolic Disorders Research Centre, Research Institute for Endocrine Sciences, Shahid Beheshti University of Medical Sciences, Tehran, Iran; ^4^Centre for Rural Health, School of Health Sciences, University of Tasmania, Tasmania, Australia; ^5^Endocrine Research Centre, Research Institute for Endocrine Sciences, Shahid Beheshti University of Medical Sciences, Tehran, Iran; ^6^Department of Community Medicine, Faculty of Medicine,Shahid Beheshti University of Medical Sciences, Tehran, Iran

**Keywords:** Cardiovascular Diseases High-Density Lipoprotein, Low-Density Lipoprotein, Survival Analysis, Longitudinal Studies

## Abstract

**Background:**

Lipid abnormalities are major risk factors of death from cardiovascular disease (CVD). As well as, lipid markers are time-dependent covariates that change with aging. Previous cohort studies have only investigated baseline measurements of lipid markers on CVD mortality.

**Materials and Methods:**

The study sample consisted of 4,148 individuals aged over 40 years. Total cholesterol (TC), LDL-cholesterol (LDL-C), and HDL-cholesterol (HDL-C) were measured in five phases. A joint model analysis was used to investigate the association between each longitudinal lipid markers and CVD mortality in men, women and pooled sample. All analysis was performed using the survival and joint modeling packages in R 3.3.3.

**Results:**

Totally, 233 CVD deaths occurred during a median follow-up of 12.4 years. For men, CVD mortality increased by 28% (confidence interval [CI]: 14%,44%) for a 10% increased in TC. For women, CVD mortality increased by 43% (CI: 22%, 68%) and 21% (CI:7%, 37%) for 10 % increase in TC and LDL-C and decreased by 18% (CI:7%, 27%) for a 10% increase in HDL-C.

**Conclusion:**

Association of lipid markers with CVD mortality is different in men and women, such that high levels of TC and LDL-C and low levels of HDL-C are risk factors of CVD mortality in women, but only TC is a risk factor of CVD mortality in men.

## Introduction

Cardiovascular disease (CVD) are the leading cause of death worldwide. An estimated 17.5 million people died from CVD in 2016 representing 31% of all global deaths. Of these, 33% are caused by CVD in lower-middle-income countries, and also the rates are rising. Similarly, the deaths caused by CVD has recently increased in Iran. In 2016, an estimated 150,000 people died from CVD in Iran, representing 41.87% of all global deaths [1].Previous cohort studies have reported a relationship between total cholesterol (TC) and low-density lipoprotein cholesterol (LDL-C) with CVD mortality[2, 3].

However, there are limited studies regarding the inverse relationship between high-density lipoprotein cholesterol (HDL-C) levels and CVD [4]. However, data on the association between lipid measures and mortality is not consistent [5].

In prospective studies, TC, LDL-C gradually decrease in the elderly. Data on the longitudinal changes of HDL-C in the elderly are relatively controversial [4].

Both TC and LDL-C increased in the elderly Iranian population [6]. Change in lipid marker over time must be considered due to its effects on death from CVD.

The majority of the studies about lipid factor effects on CVD mortality have been investigated using baseline measurements [5, 7-11]. Furthermore, few studies are addressing the effect of lipid change on CVD outcomes[12-16]. Effect of lipid changes from baseline measurement on incident coronary heart disease (CHD) has been investigated in the Tehran Lipid and Glucose Study (TLGS) population [12].

To investigate the effect of the time-varying markers on time to an event, it is necessary to use both the lipid marker trajectory over time and survival time model simultaneously. To the best our knowledge, there is no study on the investigation of lipid markers relationship with CVD mortality by considering changes in lipids over follow-up. Therefore, this study was done to evaluate the effects of TC, LDL-C, and HDL-C on CVD mortality in a cohort of the Iranian population.

## Materials and Methods

### Study Population

This study was part of the TLGS, a population-based cohort study initiated in 1999-2001 consisted of 15,010 residents (aged > 3 years old) in 13 districts of Tehran (Iran), were selected by multi-stage stratified cluster random sampling [17].

Of this population, 5089 subjects who were at least 40 years old at the cross-sectional phase of TLGS entered into the study. Subjects with missing lipid markers at baseline and follow-up visits were excluded (n=582). The latest study sample was 4507 subjects (2491 women and 2016 men). This study approved by the Ethics Committee of Research Institute for Endocrine Sciences (IR.SBMU.Endocrine.REC.1396.395) and the informed consent of all subjects was taken prior to participation in the study.

### Clinical and Laboratory Measurements

Data were collected by a trained interviewer a pretested-questionnaire, which included demographic data, past medical history, family history, consumption of antihypertensive and lipid-lowering drugs and smoking behavior. Blood samples were obtained after 12-14 hours of overnight fasting and were centrifuged within 30–45 min of collection. Fasting plasma glucose (FPG), TC, and HDL-C were assayed. The standard 2-hours post challenge plasma glucose test was conducted for those who were not on any glucose-lowering drugs. Blood pressure (BP) was measured twice on the right arm using a standardized mercury sphygmomanometer (calibrated by the Iranian Institute of Standards), and the mean of both measurements was considered as the participant’s BP. Anthropometric measures including weight and height were recorded by shoes removed and the participants by weared light clothing. Weight was measured using digital scales and recorded to the nearest 100 gram. Height was measured using a tape meter in a standing position. Body mass index (BMI) was calculated as weight (kilograms) divided by height (meters) squared. Diabetes was deﬁned as taking antidiabetic medication or FPG ≥ 7 mmol/L (126 mg/dl)[18].

### Follow-Up and Event Outcome

The follow-up was defined from February 1999 until March 2013. Lipid markers and other time-independent covariates were measured every three years until March 2013 during a median follow-up of 12.4 years. In this study, lipid markers that have been measured every three years were considered as the longitudinal response (1 up to 5 measurements were available for each subject), whereas time until CVD death was considered as the survival response. Details of the outcome data collection had been described previously [19].

### Statistical Analysis

The mean ± standard deviation or frequency of independent covariates at baseline and follow-up period were calculated. In the primary analysis, changes in covariates over follow-up were studied using linear mixed effect models in each gender separately. The joint modeling method was used to study the change in lipid markers over follow-up and effect of these changes at the time of death from CVD, simultaneously. A separate analysis was performed for each lipid markers in men, women and pooled sample after adjustment for the effect of diabetes, lipid-lowering drugs, smoking status, BMI, and systolic BP. Most of the CVD cohort studies (e.g., Framingham and TLGS), collect time-to-event variable (time to death from CVD) and repeated measurements of time-dependent variables (lipid markers). The separate analysis of time-to-event variables (survival variable) using Cox proportional hazards and time-dependent variables (longitudinal variables) using linear mixed models do not consider the association between these two variables.Joint modeling of longitudinal and survival variables is a method that considers the association between a longitudinal variable and survival variable. Another property of longitudinal and survival joint model is that it can handle measurement error of longitudinal variable [20]. In this analysis, the true value of a longitudinal variable, estimated using the observed value of the longitudinal variable, and an error term ; as presented in eq.1 [20, 21]. In joint modeling package, the true value of the longitudinal variable, was entered into survival sub-model. The coefficient of parameter in eq.2 shows an estimate of longitudinal variable effects on survival variable. denotes the relative increase in the risk of an event at time t for a one-unit increase in longitudinal variable at the same time point [21, 22]. Given that the joint model uses more data, estimated parameters are more efficient than estimates of each models parameters separately [21].

(1) $$\begin{array}{l} y_{i}(t)=m_{i}(t)+\varepsilon_{i}(t)\;\varepsilon_{i}(t)∼N(0,\sigma^{2})\\ m_{i}(t)=x_{i}^{T}(t)\beta +z_{i}^{T}(t)b_{i}\;bi∼N(0,D) \end{array}$$

(2) $$hi(t\left|m_{i}(t),w_{i}\right.)=h0(t)\exp\left\{\gamma^{T}w_{i}+am_{i}(t)\right\},t>0$$

In this study, due to the non-normal distribution of markers, logarithm-transformation of the markers were used [21]. In longitudinal sub-model, true values of each of longitudinal covariates, TC, LDL-C, and HDL-C were estimated using observed values of these covariates and other covariates including systolic BP, BMI, diabetes, smoking status, lipid-lowering drugs, and age. The logarithm of the estimated value of lipid markers and independent covariates including smoking status, lipid-lowering drugs, diabetes, BMI, and systolic BP were entered into the survival sub-model. The relative increase in the risk of CVD mortality was calculated for a 10% change in the lipid markers. The analysis was performed in both sex-adjusted and sex-specific analysis. The proportionality of the multivariable Cox model was assessed using the Schoenfeld’s test of residuals (P> 0.1). All analyses were performed using R; packages survival and joint modeling (version 3.3.3).

## Results

**Figure 1:**
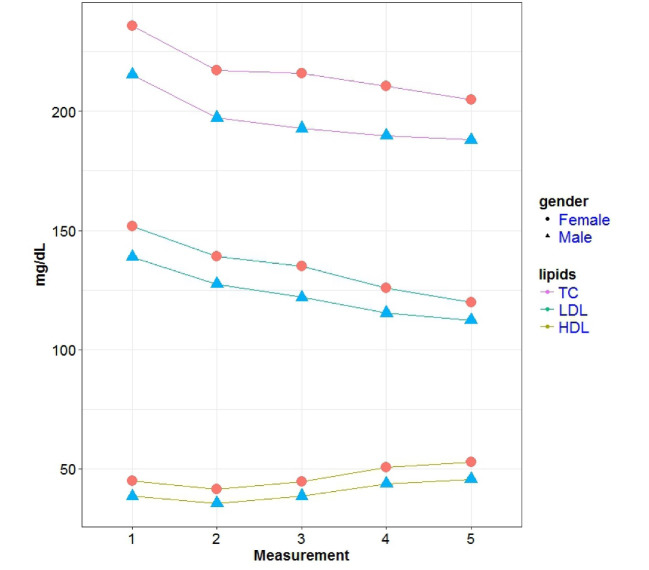
Trends of longitudinal lipid markers in five phases of the study based on gender

The study sample consisted of 4507 individuals in which 2491 were women. During follow-up of 12.4 years, 244 CVD deaths (81 women and 152 men) occurred. Table-1 illustrates the baseline and follow-up characteristics in men and women. As presented in Table-1, the univariate analysis shows that the mean of BMI and systolic BP, the proportion of subjects that consume the lipid-lowering drugs and the proportion of subjects that had diabetes increased significantly in both genders. Moreover, the proportion of smokers decreased significantly in both genders (Table-1). Comparison of characteristics showed a significant difference between male and female (Table-1). Figure-1 shows the trajectory of the mean level of lipid markers for men and women separately. As depicted in this figure, the mean level of TC and LDL-C decreased, but the mean level of HDL-C increased in both genders. The decrease of TC and LDL-C and the increase of HDL-C were significant in both sexes (Table-2). Also, the mean of lipid markers in women were higher than men (Table-2). Table-3 shows the results of the joint model. In the sex-adjusted analysis, it was observed that the relative risk of CVD mortality increased by 30% (confidence interval [CI]: 18%, 43%) and 17% (CI: 9%, 25%), respectively with a 10% increase in the TC and LDL-C levels. However, change in the HDL-C levels was not associated with CVD mortality (P=0.29). In the sex-specific analysis, it was observed that the relative risk of CV mortality increased by 28% (CI: 14%, 44%) in men and by 43% (CI: 22%, 68%) in women for a 10% increase in the TC levels. In women, the relative risk of CVD mortality increased by 21% (CI: 7%, 37%) for 10% increase in LDL-C levels and decreased by 18% (CI: 7%, 27%) for a 10% increase in the HDL-C levels. However, there was no significant relationship between CVD mortality and increase in the LDL-C (P=0.23) and HDL-C (P=0.75) levels in men.

## Discussion

In this study, we investigated the association between change in lipid markers and CVD mortality in Iranian population. At first, the true values of lipid markers estimated using observed values of lipid markers and other independent covariates, and then the effect of estimated values of lipid markers investigated on time to CVD mortality. We observed that with aging, TC and LDL-C decrease and HDL-C increase. As well as, we observed that increasing TC level increases the risk of CVD mortality in both gender, but the association between LDL-C and HDL-C depends on gender. There are few studies that investigated the association between lipid changes and CVD [13-16, 23]. Nejat *et al*. studied the association between the incident of CHD and lipid markers changes between first and second measurement in Iranian, but we investigated the effects of change in lipid markers in five measurements on CVD mortality. This study showed that an increase in TC levels was associated with an increase in CVD mortality in men and women. This result was in accordance with studies on TLGS and Framingham study [12, 15]. Generally, linear association assumed between TC and CHD and CVD mortality, e.g., in the study of Cai *et al*. CHD mortality and CVD mortality increased linearly with TC in Polish, Russian, and USA men, although there was no relationship in women [24]. However, this association was not universal. U-shaped association observed in the Korean population, i.e., groups with the lowest as well as the highest cholesterol had higher CVD mortality [25]. In the American Indian population, although a linear association has been reported between TC and CVD mortality in person without diabetes, a U-shaped association has been reported in persons with diabetes [26]. There was also a J-shaped association among Russian men [27]. HDL-C is known as a protective factor for CVD outcomes [28]. Such a protective effect has also been observed in studies that considered lipid marker changes [13, 14, 16, 23]. In the present study, HDL-C was associated with CVD mortality in women but not associated with men and sex-adjusted analysis. In Nejat *et al*. study, no significant association was observed in men and women [12]. Although LDL-C was routinely referred as a causal agent to provide CVD [27], however, in a systematic review of nine cohort studies on elderly people, no association was found in seven cohorts, and in two cohorts, high LDL-C was inversely associated with CVD mortality [29]. Nejat *et al*. observed no significant association in both gender, but in the current study [12], the association was significant in women. The distinctive properties of the present study with other studies was that majority of studies have been conducted on baseline measurements of lipid markers on the incidence of CVD, CHD, and stroke [5, 7, 8, 10, 11]. In this study, it observed that the mean of TC and LDL-C decrease, but the mean of HDL-C increase over follow-up in both sexes. A similar finding has been reported in previous studies on the TLGS population [6]. As well as, adiposity levels and prevalence of diabetes increased among the TLGS population during follow-up in both sexes. All these changes result in an increased risk of CVD [6]. Failure to consider the changes in lipid markers with aging was a limitation of these studies. The first difference of present study with Nejat *et al*. research was that we considered the effects of lipid markers change over follow-up on CVD mortality by simultaneously modeling of lipid markers change and time of CVD death. The second difference was that we handled the measurement error of lipid markers. Masudi *et al*. showed that considerable underestimation occurs due to measurement error in studying the association between lipid markers with CVD outcome [30]. In this study, CVD mortality was highly associated with lipid markers in women. It might be due to higher TC, LDL-C, and HDL-C in women. As well as, diabetes was higher in women than in men. Previous studies showed an association between type-2 diabetes and high prevalence of dyslipidemia‌, hypertension, obesity [31], and CVD outcome [26]. Higher association between lipid markers and CVD mortality in women also reported in other studies, e.g., Lipid Research Clinics Prevalence Mortality Follow-up Study (LRCF)[32]. For the first time, our study investigated lipid markers association with CVD mortality in joint modeling framework. It is required to consider some limitations of this study. Firstly, lipid levels at each time point were determined based on a single measurement. Secondly, given that the study sample was selected from Tehran’s urban population, extrapolation of findings might be difficult for the total population. Thirdly, we used a modiﬁed Friedewald equation for LDL-C calculation, which might not be as accurate as the direct assessment.

## Conclusion

Changes of lipid markers over time is similar in men and women; but the association of lipid markers with CVD mortality is different in men and women, such that high level of TC and LDL-C, and low level of HDL-C increase CVD mortality in women, but only TC is a risk factor of CVD mortality in men.

## Acknowledgment

This article has been extracted from the thesis of Mr. Bagher Pahlavanzade in the School of Allied Medical Sciences of Shahid Beheshti University of Medical Sciences. We would like to express our appreciation to TLGS participants and research team members for their contribution to the study. This research did not receive any specific grant from funding agencies in the public, commercial, or not-for-profit sectors.

## Conflict of Interest

The authors declare that there is no competing of interests.

**Table 1:** Change of Characteristics of Participants at Five Phases (Baseline + 4 Follow-Ups)

		**1** ^st^ ** phase**	**2** ^nd^ ** phase**	**3** ^rd^ ** phase**	**4** ^th^ **phase**	**5** ^th^ ** phase**	**Change in each year** ^a^	**P-value** ^b^
**SBP**	Male Female	126.6(20.6) 128.5(21.1)	126.4(20.2) 126.6(20.7)	127.9(20.6) 125.2(20.4)	129.3(19.7) 128.8(21.6)	129.8(19.7) 130(21.6)	0.037(0.03,0.04)^***^ 0.02(0.016,0.03)^***^	<0.001
**BMI**	Male Female	26.4(3.8) 29.2(4.5)	26.8(3.7) 30(4.6)	26.8(3.8) 30.2(4.8)	27(3.8) 30.8(5.1)	26.9(4.1) 30.8(5.1)	0.029(0.019,0.38)^***^ 0.12(0.11,0.13)^***^	<0.001
**Smoking**	Male Female	367(22) 51(2.3)	194(19.3) 30(1.8)	165(13.8) 28(1.7)	179(13.8) 32(1.8)	147(12) 23(1.4)	6.1(5.3,7)^***^ 4(1,7)^***^	<0.001
**Diabetes**	Male Female	202(11.4) 336(14.5)	169(13.4) 241(16.1)	172(13.1) 296(16.5)	227(17.2) 368(20.6)	236(19) 335(20.2)	3.6(2.4,4.8)^***^ 3(2.1,4.1)^***^	<0.001
**Lipid-lowering drugs**	Male Female	68(4) 184(8.4)	56(4.7) 163(10)	93(7.8) 231(14.6)	192(14.8) 425(24.5)	266(21.8) 553(34)	16(14,18)^***^ 14(0.13,15)^***^	<0.001

Data are shown as mean (SD) for continuous variables or frequency (%) for categorical variables. **SBP:** Systolic blood pressure; **BMI:** Body mass index *** P-value for change during follow-up were smaller than 0.01 ^a^Test for a linear trend according to linear regression for continuous variables and for categorical variables ^b^For comparison between male and female

**Table 2:** Change of TC, LDL-C, and HDL-C At Five Phases (Baseline + 4 Follow-Ups)

		**1** ^st^ ** phase**	**2** ^nd^ ** phase**	**3** ^rd^ ** phase**	**4** ^th^ **phase**	**5** ^th^ ** phase**	**Change in each year** ^a^	**P-value** ^b^
**TC**	Male	215(42.6)	197.1(38.6)	192.8(36.5)	189.7(40.8)	188(39.6)	-2.12(-2.28, -1.95)^***^	<0.001
	Female	235.7(48.7)	217.2(42)	215.8(39.5)	210.5(43.1)	205(41.6)	-2.26(-2.43, -2.08)^***^	
**LDL-C**	Male	138.8(35.7)	127.3(32.3)	121.9(31.8)	115.3(33.6)	112.4(34.8)	-2.01(-2.14, -1.87)^***^	<0.001
	Female	151.6(40)	139.1(35.7)	134.9(33.7)	125.6(38.2)	120(38.1)	-2.34(-2.48, -2.19)^***^	
**HDL-C**	Male	38.4(9.4)	35.5(8.8)	38.5(8.6)	43.6(9.7)	45.5(10.3)	0.6(0.58,0.62)^***^	<0.001
	Female	44.7(11.3)	41.4(10.4)	44.5(10.3)	50.6(11.2)	52.7(12.4)	0.67(0.65,0.69)^***^	

Data are shown as mean **(SD)** for continuous variables or frequency (%) for categorical variables. **TC:** Total cholesterol; **HDL-C:** High-density lipoprotein cholesterol; **LDL-C:** Low-density lipoprotein cholesterol ***P-value for change during follow-up were smaller than 0.01 ^a^Test for a linear trend according to linear regression for continuous variables ^b^For comparison between male and female

**Table 3:** Relative Risks of Lipid Markers on CVD Mortality in Total Sample and Each Gender

		**Relative Risks** ^c^		
		**Estimates**	**Confidence Interval (95%)**	**P-value**
**Male** ^a^	TC LDL-C HDL-C	1.28 1.04 1,01	1.14, 1.44 0.96, 1.13 0.92, 1.1	<0.001 0.23 0.75
**Female** ^a^	TC LDL-C HDL-C	1.43 1.21 0.82	1.22, 1.68 1.07, 1,37 0.73, 0.93	<0.001 0.002 0.002
**Total** ^b^	TC LDL-C HDL-C	1.3 1.17 1.03	1.18, 1,43 1.09, 1.25 0.96, 1.11	<0.001 <0.001 0.29

**TC:** Total cholesterol; **HDL-C:** High-density lipoprotein cholesterol; **LDL-C:** Low-density lipoprotein cholesterol ^a^ Model was adjusted for diabetic status, smoking, lipid-lowering drugs, the first measurement of systolic blood pressure, and body mass index. ^b^Model was adjusted for sex, diabetic status, smoking, lipid-lowering drugs, the first measurement of systolic blood pressure, and body mass index. ^c^ values of relative risk from the joint model for 10% increase.
